# Mitochondrial pyruvate carrier 2 mitigates acute kidney injury via sustaining mitochondrial metabolism

**DOI:** 10.7150/ijbs.98627

**Published:** 2024-08-19

**Authors:** Lin Wu, Qing Li, Fang Lu, Li Qian, Ying Pan, Chen Chen, Zhimin Huang, Suyan Duan, Bo Zhang, Hongwei Liang, Changying Xing, Huijuan Mao, Yanggang Yuan

**Affiliations:** 1 Department of Nephrology, the First Affiliated Hospital of Nanjing Medical University, Nanjing Medical University, Nanjing, China.; 2 School of Life Science and Technology, China Pharmaceutical University, Nanjing, Jiangsu, China.

**Keywords:** cisplatin, acute kidney injury, metabolism reprogramming, MPC2, artemether

## Abstract

Cisplatin, a chemotherapeutic drug, can result in acute kidney injury (AKI). Currently, there are no effective prevention methods. An incomplete understanding of the pathogenesis of AKI is a major barrier to the development of effective therapies. Metabolism reprogramming shift to glycolysis was involved in AKI pathogenesis. Glycolysis results in the pyruvate production. The mitochondrial pyruvate carrier (MPC) conveys cytosol pyruvate into mitochondria, promoting the tricarboxylic acid cycle. In this current study, we found a reduction in MPC2 expression in mice and cultured HK2 cells with cisplatin-induced AKI. MPC2 overexpression attenuated cisplatin-mediated nephrotoxicity both *in vitro* and *in viv*o via restoring pyruvate metabolism and mitochondrial function. Knockdown of MPC2 reversed this effect. Furthermore, artemether, an MPC2 potential activator, could mitigate AKI via regulating MPC2-mediated pyruvate metabolism. Our findings revealed that MPC2-pyruvate metabolism axis was a promising strategy to alleviate AKI induced by cisplatin.

## Introduction

Acute kidney injury (AKI) takes a high risk of morbidity and mortality^1^. Acute tubular necrosis (ATN) is one of the main structural changes of AKI. ATN can occur in response to ischemia, exposure to toxins, or sepsis. Nephrotoxicity is a serious adverse effect of the chemotherapeutic drug cisplatin^2^, ^3^. Cisplatin results in AKI by targeting the renal tubular epithelial cells. Damaged proximal tubular cells undergo alterations in cellular metabolism and cell cycle^4^, ^5^. However, there is to date no specific drug for AKI, whose improvement is urgent.

Due to the high metabolic demand of the kidneys, energy metabolism is particularly important for cellular health and function. The kidney exhibits a high oxygen consumption state to satisfy the energy needs of tubular reabsorption^6^. The energy of renal tubular epithelial cells needed is provided by fatty acid β-oxidation (FAO), as well as oxidative phosphorylation (OXPHOS)^7^. Emerging evidence suggests that metabolic reprogramming is a key event contributing to AKI. A short period metabolic reprogramming away from FAO-driven OXPHOS to glycolysis compensates for impaired mitochondrial ATP generation and exhibits partial renal-protective properties. However, its long-term outcome of AKI is not promising^8^. Therefore, understanding the specific metabolic pathways that are associated with protection during AKI is a valuable approach to identify potential drug targets.

The mitochondrial pyruvate carrier (MPC) resides at a central position via importing pyruvate to the mitochondrial matrix from the cytosol, connecting the link reaction between glycolysis and OXPHOS^9^. The MPC of mammals consists of two important and interdependent subunits, MPC1 and MPC2. The absence of any of them results in abnormalities in the transport of pyruvate and has a major influence on the cellular metabolic status^10^. A recent study suggested that tubular cell-specific MPC1 knockout mice showed increased glycolysis and decreased oxidative stress and kidney injury in an experimental rhabdomyolysis-induced AKI model^11^. However, although MPC1 and MPC2 have similar structures and functions, they are not identical in cell outcomes reported in different literature^12^. The specific role of MPC2 in acute kidney injury is not yet clear.

Our current study verified the importance of MPC2 in combating cisplatin-induced AKI by regulating pyruvate metabolism in animal models and proximal renal tubular cells. These results provided a potential method for protecting against cisplatin nephrotoxicity.

## Results

### Metabolic alterations in HK2 cells induced by cisplatin

The tricarboxylic acid cycle can be driven by three sources including pyruvate, fatty acids, and glutamine/glutamate^13^. To investigate the diverse substrates utilization for mitochondrial respiration, HK2 cells were incubated with UK5099 (a mitochondrial pyruvate carrier inhibitor), Etomoxir, (a carnitine palmitoyltransferase-1 inhibitor), and BPTES (a selective glutaminase inhibitor) while monitoring oxygen consumption rate (OCR) (Supplemental Figure S1A). OCR measurements were conducted with the serially addition of oligomycin (ATP synthase inhibitor), FCCP (uncouple ATP synthesis), and a mixture of antimycin A (an inhibitor of Complex III) plus rotenone (a specific inhibitor of Complex I), yielding bioenergetic information about basal, ATP production, maximal, and nonmitochondrial respiration OCR, respectively (Supplemental Figure S1B). As shown in Supplemental Figure S1C-H, we observed that each specific treatment influenced basal respiration, maximum respiration, and ATP production OCR, which was inhibited by cisplatin. Moreover, OCR under cisplatin treatment was further reduced by blocking the utilization of pyruvate or fatty acids. However, no difference in OCR was anymore detected by inhibiting the glutamine route. Overall, these data indicated that cisplatin impaired the utilization of these fuels by mitochondria and HK2 cells relied on pyruvate and fatty acids to fuel metabolism under cisplatin treatment.

### MPC2 expression is decreased in AKI

To explore the expression pattern of key regulators of pyruvate and fatty acids metabolism in AKI, the transcriptional level of MPC and carnitine palmitoyltransferase (CPT) was analyzed firstly via GEO database (accession: GSE52004 and GSE106993). RNA-sequencing (RNA-seq) analyses showed a reduction in MPC2 and CPT1b RNA levels in the ischemia reperfusion-induced tubular epithelial cell injury and a reduction in MPC1, MPC2, CPT1a, and CPT2 RNA levels in the cisplatin-induced models (Supplemental Figure S2). Consistently, Single Cell RNA Sequencing Data also showed a reduction in MPC2 RNA levels in both cisplatin and ischemia reperfusion-induced tubular epithelial cell injury (Supplemental Figure S3).

**Figure 1:**
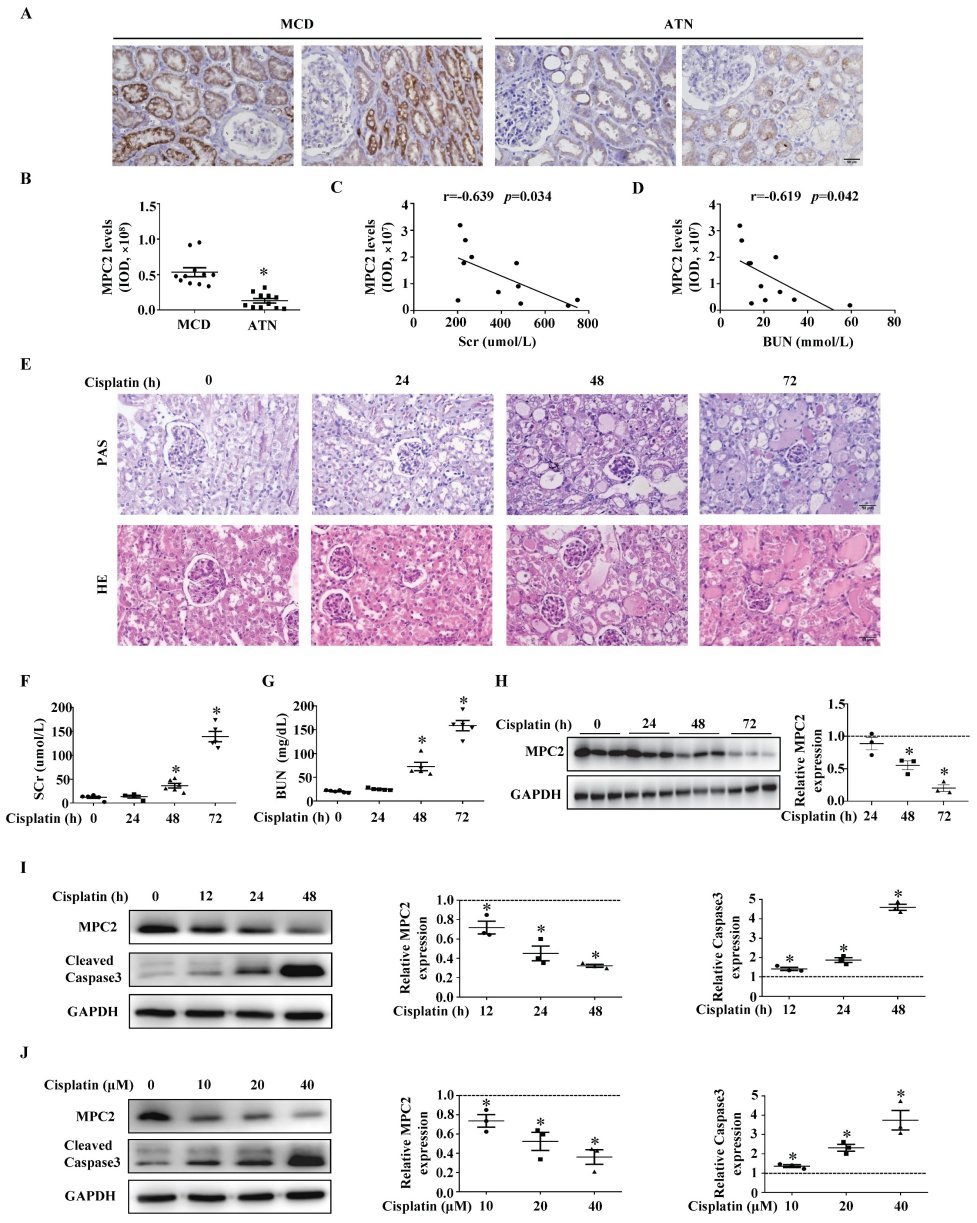
** MPC2 was downregulated in the kidneys of AKI patients, cisplatin nephrotoxicity murine models, and HK2 cells treated with cisplatin.** (A) Immunohistochemical observation of MPC2 contents in biopsy samples from minimal change disease (MCD) and acute tubular necrosis (ATN) patients. Scale bar=50 μm. (B) Quantifications of MPC2 expression in MCD and ATN patients. Pearson correlation analysis of MPC2 levels and serum creatinine (SCr) levels (C), blood urea nitrogen (BUN) levels (D) of 11 hospitalized ATN patients. (E) HE and PAS staining of renal tissues in corresponding groups. Scale bar=50 μm. The serum concentrations of SCr (F), and BUN (G) were measured for the indicated periods after cisplatin treatment. Data are presented as means ± SEM (n=5-6). *P < 0.05 vs. control. (H) Immunoblot and semiquantification of MPC2 in kidneys of cisplatin treatment mice. Data are means ± SEM (n=3), *P < 0.05 vs. control group. (I) Left: Western blot detection of MPC2 and cleaved caspase3 in HK2 cells with cisplatin treatment at different points in time. Right: Densitometric analysis. (J) Left: Western blot detection of MPC2 and cleaved caspase3 in HK2 cells with cisplatin treatment at different doses. Right: Densitometric analysis. Data are shown as means ± SEM repeated three times. *P < 0.05 vs. control group.

MPC2 protein expression was decreased in renal tissues of AKI patients (Figure 1A and 1B). The MPC2 protein levels showed a negative correlation with SCr (Figure 1C) and BUN levels (Figure 1D).

The expression of MPC2 was then verified in AKI mice kidneys induced by cisplatin. HE and PAS staining demonstrated that cisplatin induced renal proximal tubular injury (Figure 1E), which was featured by the brush border loss, tubular dilatation, and cast formation. SCr (Figure 1F) and BUN (Figure 1G) significantly increased in mice 48 h after cisplatin injection. Western blot indicated that MPC2 protein levels markedly decreased from day 2 to day 3 compared to controls (Figure 1H). Moreover, in cultured HK2 cells, along with the activation of the caspase3, MPC2 expression was reduced by cisplatin in a time- (Figure 1I) and dose-dependent manner (Figure 1J).

### MPC2 overexpression attenuates AKI induced by cisplatin

**Figure 2:**
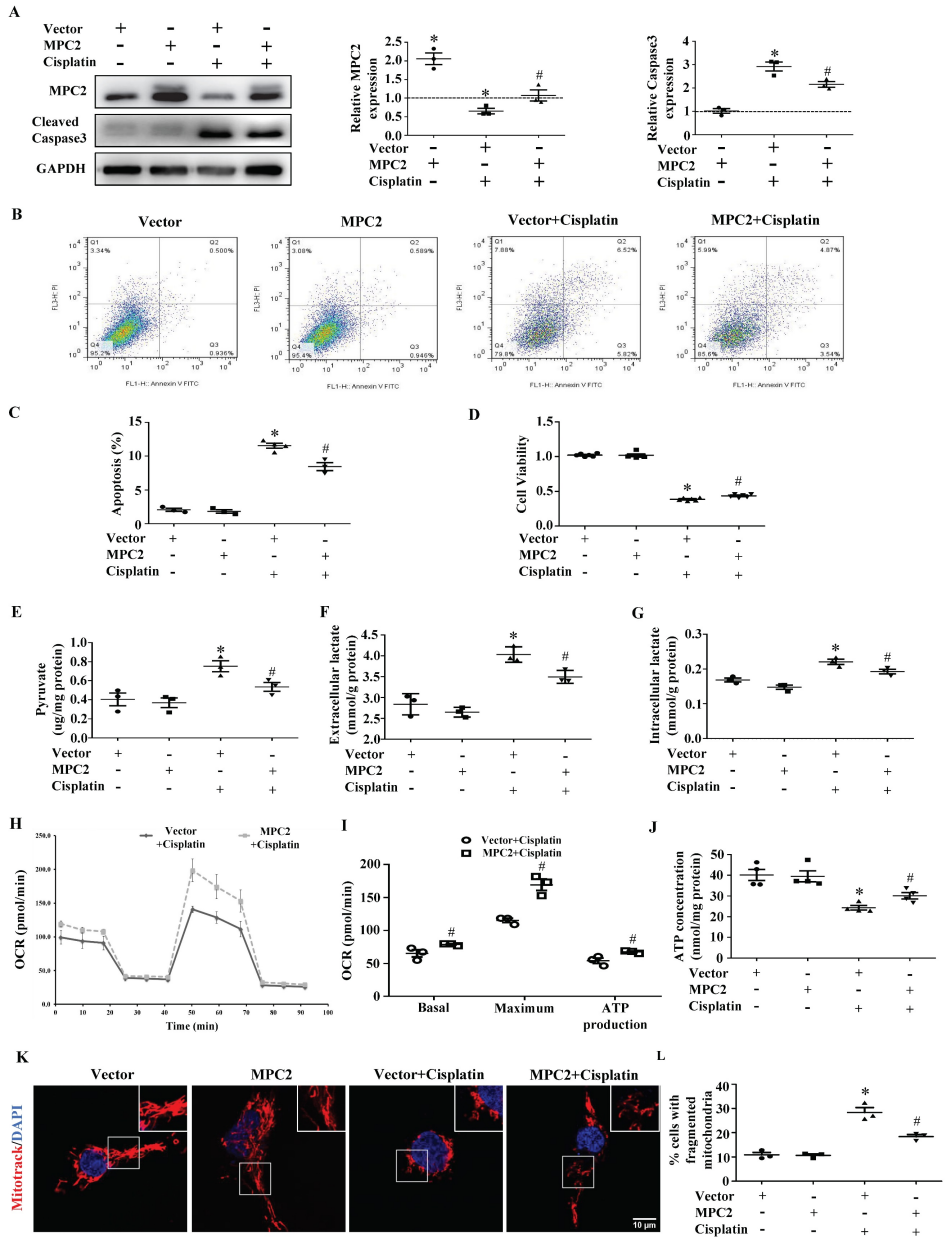
** Effect of MPC2 overexpression on HK2 cells damage and mitochondrial impairment induced by cisplatin.** HK-2 cells were transfected with empty vector or with plasmid encoding MPC2, and then incubated with cisplatin (40 μM) for 24 hours. (A) Immunoblotting analysis. Left: representative western blots. Right: densitometric analysis. (B) Representative images of cell apoptosis determined by flow cytometry. (C) Statistical results. (D) Cell viability analysis by CCK8 kit. (E) Pyruvate levels in cells were analyzed and normalized to protein levels. (F) Extracellular lactate levels normalized to protein levels. (G) Intracellular lactate levels normalized to protein levels. (H) Measurement of OCR using an XF96 Extracellular Flux Analyzer. (I) OCR for basal respiration, maximal respiration, and ATP production. (J) ATP contents. (K) MitoTracker Red staining. Scale bar=10 μm. (L) Quantification of mitochondrial morphology. Data are shown as means ± SEM repeated three times. *P < 0.05 vs. control. *#P* < 0.05 vs. cisplatin treatment group. Vector, control, MPC2, MPC2 overexpression.

To further verify the effects of MPC2 on AKI, MPC2 was overexpressed in HK2 cells before cells were subjected to cisplatin. MPC2 overexpression prevented cisplatin-induced caspase3 activation (Figure 2A). Also, apoptosis quantified by flow cytometry (Figure 2B and 2C) and CCK-8 (Figure 2D) assay revealed that MPC2 overexpression significantly decreased HK2 cell injury. The above results showed that the upregulation of MPC2 provided a protective effect on HK2 cells treated with cisplatin. In order to verify the role of MPC2 in the metabolic process, we tested the pyruvate and lactate levels upon exposure to cisplatin. As shown in Figure 2E, the intracellular pyruvate was increased under cisplatin treatment and MPC2 overexpression inhibited the effects of cisplatin. Also, MPC2 overexpression prevented the increase of extracellular (Figure 2F) and intracellular (Figure 2G) lactate levels. Taken together, these data demonstrated that MPC2 overexpression protected against HK2 cell injury exposure to cisplatin via inhibiting glycolytic efflux.

### MPC2 overexpression attenuates cisplatin-induced mitochondrial dysfunction

In order to further unlock the metabolic reprogramming status, functional mitochondria analyses were measured with a seahorse XFe96 analyzer. As shown in Figure 2H and 2I, the decreased basal, maximal, and ATP production OCR in cisplatin-treated HK2 cells were reversed after MPC2 overexpression. Consistently, MPC2 overexpression prevented cisplatin-induced reduction in ATP production (Figure 2J). Previous study showed that changes in this energy generation process could impact the morphology of the mitochondria. Compared with the control group, MPC2 overexpression inhibited cisplatin-induced mitochondrial fission (Figure 2K and 2L). These studies suggested a vital role for MPC2-mediated pyruvate import in mitochondrial function.

### MPC2 knockdown aggravates cisplatin-induced AKI

**Figure 3:**
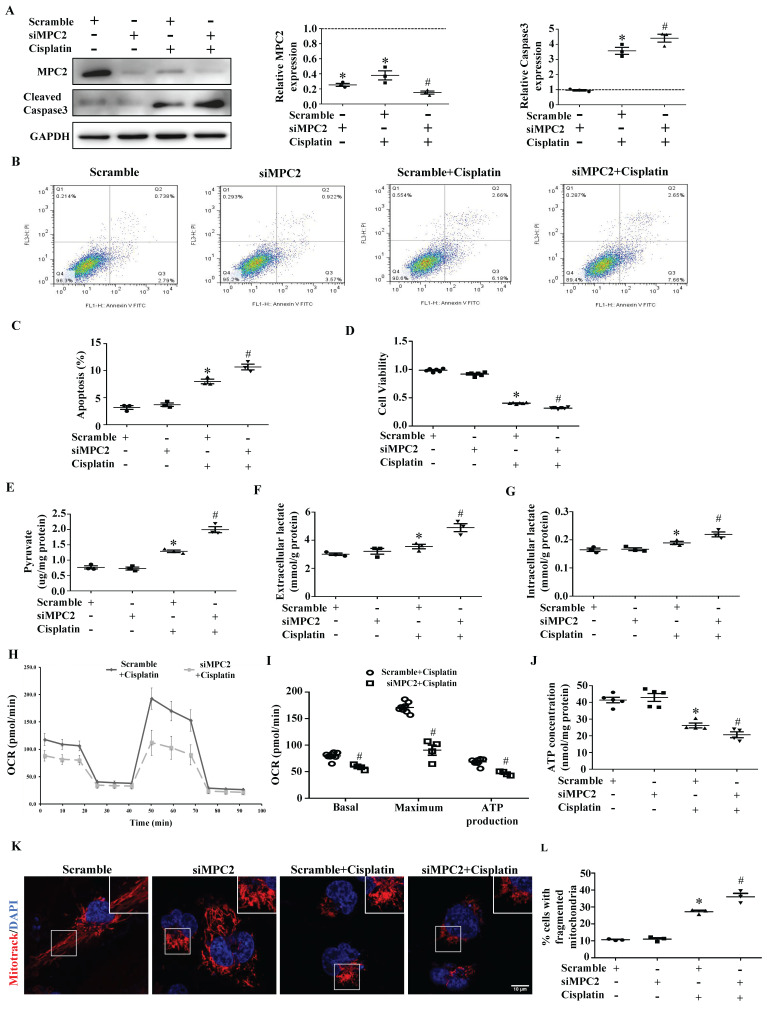
** Effect of MPC2 siRNAs on HK2 cells damage and mitochondrial impairment induced by cisplatin.** HK-2 cells were transfected with MPC2 siRNA followed by cisplatin (40 μM) treatment for 24 hours. (A) Immunoblotting analysis. Left: representative western blots. Right: densitometric analysis. (B) Representative images of cell apoptosis determined by flow cytometry. (C) Statistical results. (D) Cell viability analysis by CCK8 kit. (E) Pyruvate levels in cells were analyzed and normalized to protein levels. (F) Extracellular lactate levels normalized to protein levels. (G) Intracellular lactate levels normalized to protein levels. (H) Measurement of OCR using an XF96 Extracellular Flux Analyzer. (I) OCR for basal respiration, maximal respiration, and ATP production. (J) ATP contents. (K) MitoTracker Red staining. Scale bar=10 μm. (L) Quantification of mitochondrial morphology. Data are shown as means ± SEM (n ≥ 3). *P < 0.05 vs. control group. *#P* < 0.05 vs. cisplatin treatment group. Scramble, control, siMPC2, MPC2 siRNA.

Compared with control siRNA-transfected cells, MPC2 knockdown (MPC2 siRNA) cells showed a lower MPC2 signal, as expected. In accordance with the findings observed upon MPC2 overexpression, we found that MPC2 knockdown aggravated cisplatin-induced HK2 cell injury, which was examined by caspase3 activation (Figure 3A), apoptosis (Figure 3B and 3C) and cell viability (Figure 3D). Also, the knockdown of MPC2 promoted the cisplatin-induced increase of pyruvate (Figure 3E) and lactate levels (Figure 3F and 3G), indicating that MPC2 knockdown deteriorated the effects of cisplatin on pyruvate transport and the lactate production.

### MPC2 knockdown aggravates cisplatin-induced mitochondrial dysfunction

The functional assay of mitochondrial respiration derived using the Seahorse analysis showed that basal respiration, maximal respiration, and ATP generation significantly dropped after the knockdown of MPC2 under cisplatin treatment (Figure 3H and 3I). Moreover, MPC2 knockdown aggravated actual ATP loss (Figure 3J) and mitochondrial fission (Figure 3K and 3L) induced by cisplatin. Taken together, these data indicated that MPC2 knockdown accumulated dysfunctional mitochondria in cells under cisplatin overload.

### *In vivo* overexpression of MPC2 inhibits cisplatin-induced AKI

**Figure 4:**
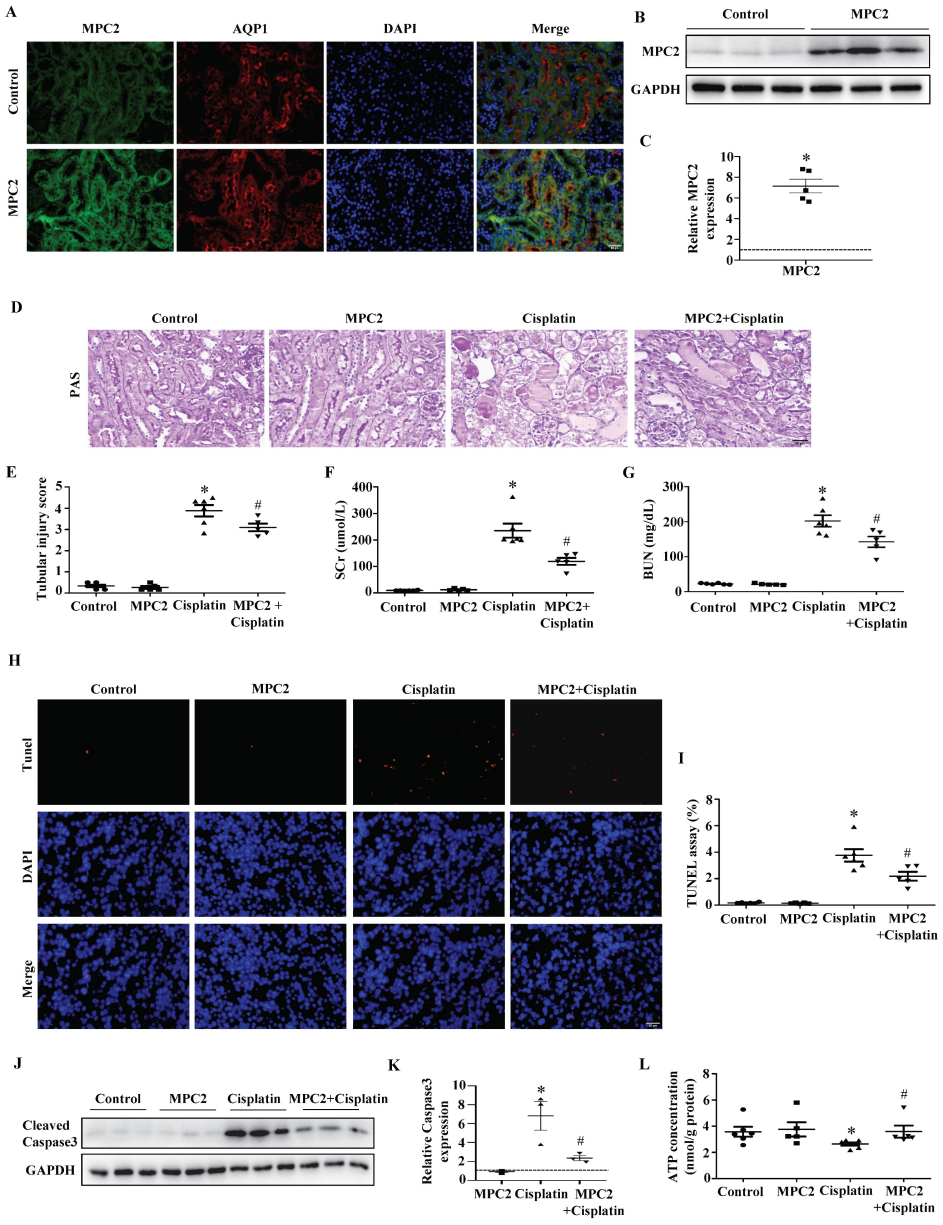
** Effect of MPC2 overexpression on cisplatin-induced AKI *in vivo*.** (A) Representative graphs of MPC2 and AQP1 immunofluorescence staining. Scale bar=50 μm. (B) MPC2 protein levels by western blot in the kidney cortex. (C) Densitometry analysis. (D) PAS staining. Scale bar=50 μm. (E) Quantitation of tubular injury. The levels of serum creatinine (F) and blood urea nitrogen (G) were examined. (H) TUNEL staining. Scale bar=50 μm. (I) Semiquantitative assessment of positive cells. (J) Representative western blots showing cleaved caspase3. (K) Densitometry analysis of the western blots. (L) ATP production. Data are shown as means ± SEM (n ≥ 3). *P < 0.05 vs. control. #*P* < 0.05 vs. cisplatin treatment group. MPC2, MPC2 Knockin.

To confirm our findings *in vivo*, we overexpressed MPC2 in mice by intravenous injection. *In vivo* plasmid transfection was performed by a lipid-based transfection kit as previously described. The overexpression of MPC2 was found highly effective in mouse renal tubular cells by immunofluorescence assay (Figure 4A). Western blotting reconfirmed the upregulation of MPC2 protein expression in kidney tissues (Figure 4B and 4C). Acute renal injury was detected by PAS staining, illustrating that overexpression of MPC2 attenuated renal pathological changes in the cisplatin-induced AKI model (Figure 4D and 4E). The kidney function of cisplatin-induced mice, reflected by SCr and BUN levels, was significantly improved after MPC2 overexpression (Figure 4F and 4G). In line with this, MPC2 overexpression was found to suppress tubular cell apoptosis in the kidneys, as revealed by the TUNEL assay (Figure 4H and 4I). Also, MPC2 overexpression inhibited cisplatin-induced caspase3 activation (Figure 4J and 4K). Moreover, MPC2 overexpression restored ATP generation in cisplatin-induced mice (Figure 4L). Together, these data found that MPC2 overexpression alleviated cisplatin-induced acute renal epithelial cell damage.

### MPC2 agonist inhibits cisplatin-induced AKI

**Figure 5:**
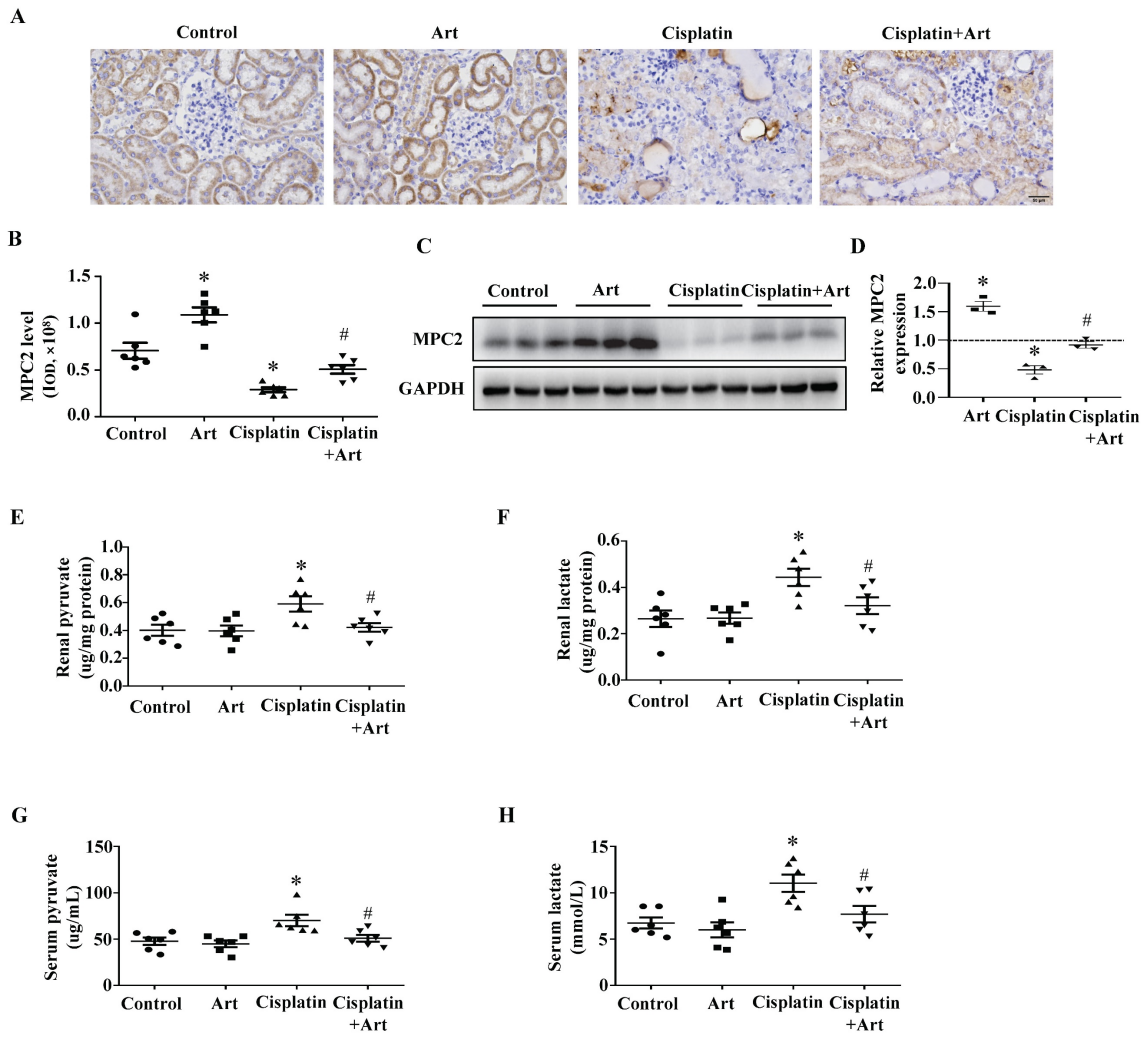
** Effect of Art on MPC2 expression under cisplatin treatment *in vivo*.** (A) Immunohistochemical observation of MPC2 expression in mice kidney. Scale bar=50 μm. (B) Quantitative analysis of MPC2 expression. (C) MPC2 protein expression by immunoblotting analysis in the kidney cortex. (D) Densitometry analysis of the western blots of MPC2. (E) Renal pyruvate levels. (F) Renal lactate levels. (G) Serum pyruvate content. (H) Serum lactate content. Data are shown as the means ± SEM (n ≥ 3). *P < 0.05 vs. control. #*P* < 0.05 vs. cisplatin treatment group. Art, artemether.

A previous study demonstrated that artemether (Art), an artemisinin derivative applied in the treatment option for malaria, could protect against kidney injury in T2D db/db mice partially via increasing MPC1 and MPC2 levels^14^. In our current study, immunohistochemistry (Figure 5A) and quantitative analysis (Figure 5B) showed Art restored MPC2 expression in cisplatin-induced AKI. Consistently, western blotting results demonstrated that Art significantly attenuated the reduction of MPC2 expression (Figure 5C and 5D). Meanwhile, Art treatment prevented cisplatin-induced both renal and serum pyruvate (Figure 5E and 5G) and lactate levels (Figure 5F and 5H).

**Figure 6:**
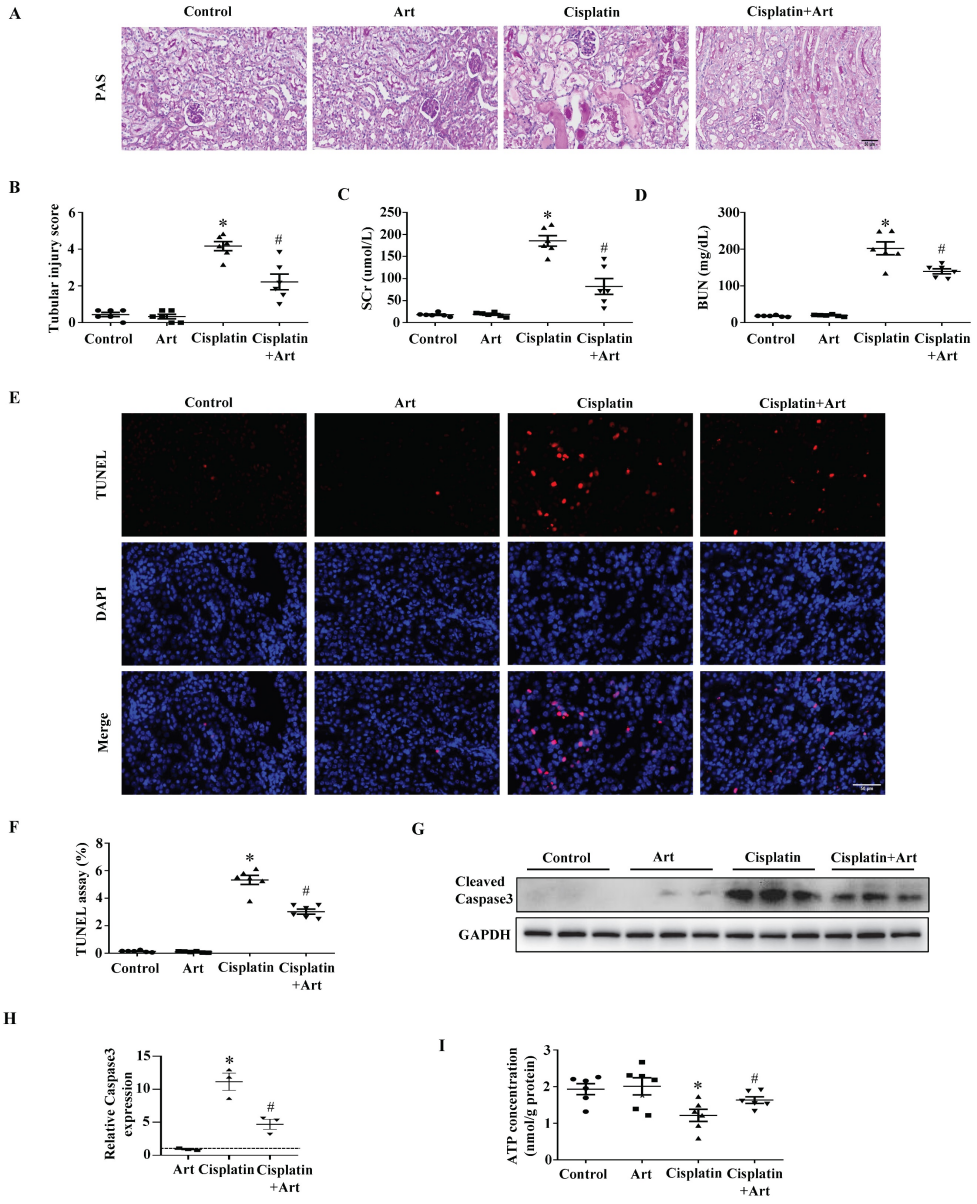
** The protective effect of Art on cisplatin-induced AKI *in vivo*.** (A) PAS staining. Scale bar=50 μm. (B) Quantitation of tubular injury. The serum concentration of (C) creatinine and (D) blood urea nitrogen (BUN). (E) Representative pictures of TUNEL staining. Scale bar=50 μm. (F) Semiquantitative assessment of positive cells. (G) Representative western blots showing cleaved caspase3. (H) Densitometry analysis of the western blots. (I) ATP contents. Data shown as means ± SEM (n ≥ 3). *P < 0.05 vs. control. #*P* < 0.05 vs. cisplatin treatment group. Art, artemether.

Then we verified the therapeutic effect of Art in AKI. As shown in Figure 6A and 6B, Art significantly reduced the tubular injury score quantified from PAS staining of kidney sections. Also, Art treatment resulted in decreases in serum kidney injury markers as determined by SCr (Figure 6C) and BUN (Figure 6D) levels. Consistently, renal tubular cell injury tested by TUNEL staining (Figure 6E and 6F) and caspase3 activation (Figure 6G and 6H) was significantly inhibited by Art treatment. In addition, Art treatment restored ATP production in cisplatin-induced mice (Figure 6I). Taken together, the results above showed that Art might attenuate AKI induced by cisplatin through affecting MPC2-mediated pyruvate metabolism.

### MPC2 agonist inhibits cisplatin-induced HK2 cell injury via upregulating MPC2 levels

**Figure 7:**
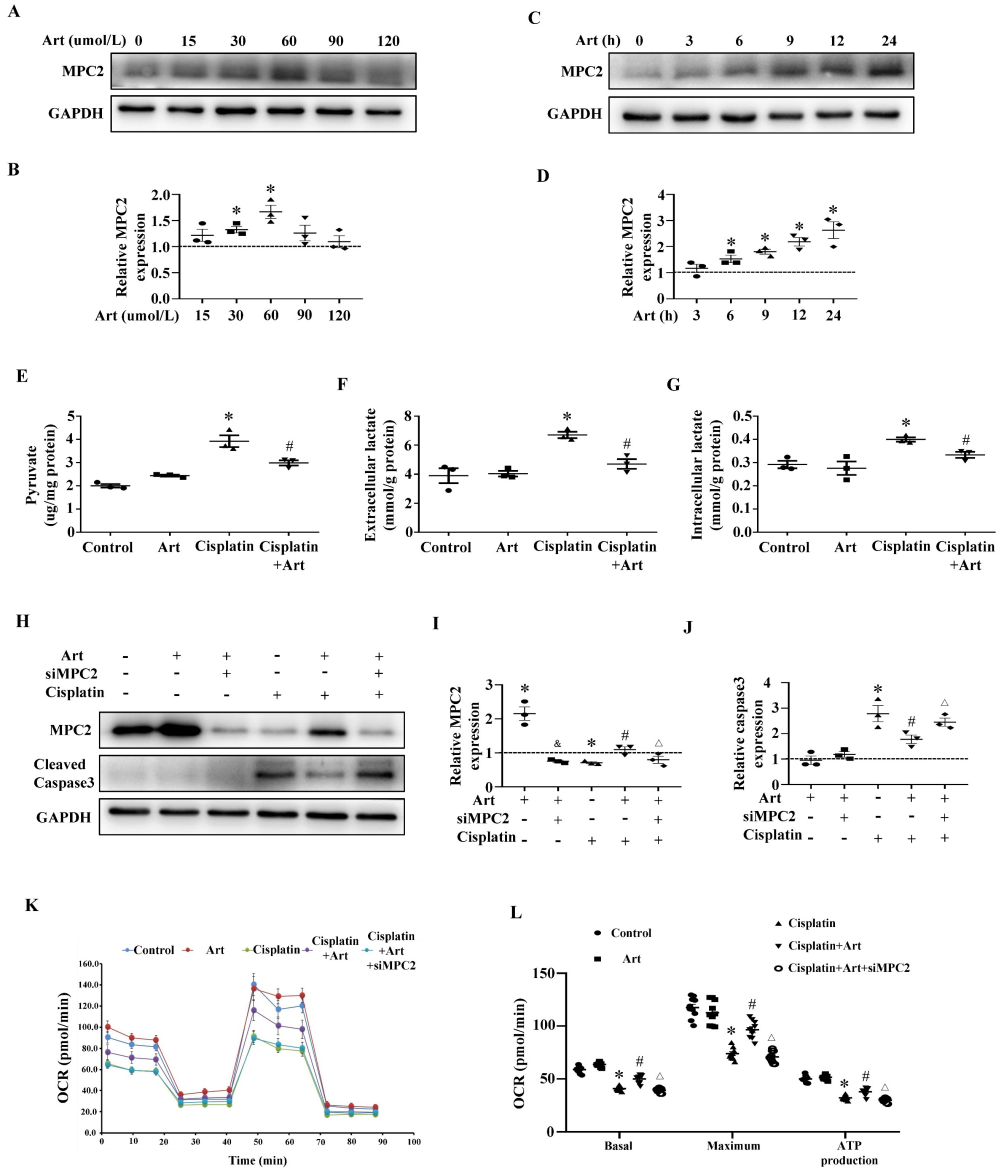
** The protective effect of Art on cisplatin-induced HK2 cell damage *in vitro*.** HK2 cells were incubated with different doses of Art (0-120 μM) for 24 h. (A) Western blots of MPC2. (B) Densitometric analysis of MPC2. HK2 cells were treated with 60 μM Art for the indicated periods. (C) Western blot analysis of MPC2. (D) Densitometric analysis of MPC2. (E) Pyruvate levels in cells were analyzed and normalized to protein levels. (F) Extracellular lactate levels normalized to protein levels. (G) Intracellular lactate levels normalized to protein levels. HK2 cells were pre-treated for 1 h with Art (60 μM), followed by incubation with cisplatin (40 μM) for a further 24 h. HK-2 cells were transfected with MPC2 siRNA for 24 h and were pretreated with Art (60 μM) followed by incubation with cisplatin for a further 24 h. (H) Representative western blots showing MPC2 and cleaved caspase3. (I) Densitometric analysis of MPC2. (J) Densitometric analysis of cleaved caspase3. (K) Measurement of OCR using an XF96 Extracellular Flux Analyzer. (L) OCR for basal respiration, maximal respiration, and ATP production. Data shown as means ± SEM (n ≥ 3). *P < 0.05 vs. control. &*P* < 0.05 vs. Art group. #*P* < 0.05 vs. cisplatin treatment group. ∆*P* < 0.05 vs. cisplatin+Art treatment group. MPC2 siRNA, Art, artemether.

To further confirm that MPC2 was involved in the protective action of Art under cisplatin treatment, we examined the function of Art on the expression of MPC2 in HK2 cells. Art dose-dependently increased MPC2 expression and the effect reached a maximum at 60 μM (Figure 7A and 7B). Moreover, the MPC2 expression was upregulated by 60 μM Art in a time-dependent manner (Figure 7C and 7D). In agreement with the previous study *in vitro,* Art treatment inhibited the production of pyruvate (Figure 7E) and lactate (Figure 7F and 7G) induced by cisplatin. We further explored the role of the Art on cell damage and mitochondrial impairment. As shown in Figure 7H, 7I, and 7J, Art restored MPC2 levels and reduced cisplatin-induced caspase3 activation. Furthermore, Art blocked the impairment of the mitochondrial respiratory function (Figure 7K and 7L). All these protective effects of Art were abolished by the siRNA knockdown experiments of MPC2. Thus, these results supported the hypothesis that Art alleviated cisplatin-induced tubular cell damage via MPC2 activation.

### FAO inhibition exacerbates cisplatin-induced metabolic rewiring in HK2 cells

Given that MPC2 reduction disrupted the mitochondrial metabolism, we examined the fuel source changes in MPC2 knockdown HK2 cells under cisplatin treatment. As shown in Supplemental Figure S4A-F, instead of glutaminase and pyruvate transport inhibition, the inhibition of CPT-dependent FAO by etomoxir significantly exacerbates the reduction of ATP generation in HK2 cells under MPC2 knockdown and cisplatin treatment. Similarly, reducing FAO by either CPT1 siRNA or CPT2 siRNA could promote the loss of energy source (Supplemental Figure S4G and S4H). These data implied that FAO might contribute to the energy source in tubular cells after MPC2-mediated pyruvate transport impairment induced by cisplatin. Consistent with previous RNA-sequencing (RNA-seq) analyses, CPT1a expression was reduced by cisplatin in a time- (Supplemental Figure S4I and 4J) and dose-dependent manner (Supplemental Figure S4K and 4L).

## Discussion

Our current study found the downregulation of MPC2 expression in renal tubular in the biopsies from ATN patients and cisplatin-induced tubular cells. MPC2, the master regulator of pyruvate flux into mitochondria, protected from tubular cell injury induced by cisplatin *in vivo* and *in vitro*. Moreover, artemether, the potential activator of MPC2, prevented cisplatin nephrotoxicity via regulating MPC2-mediated pyruvate metabolism. Based on these findings, MPC2 in renal tubular cells is a potential therapeutic target and artemether may become a promising drug for cisplatin-induced AKI.

FAO may be the preferred energy substrate for tubular epithelial cells to meet high energy needs. Kidney tubules have the second highest mitochondrial density after the heart^15^. Previous studies showed that renal tubular epithelial cells undergo metabolic reprogramming during AKI with a shift from FAO to glycolysis^16^. Literature indicate that turning to aerobic glycolysis may be injurious to tubular epithelial cells. Thus, it is possible that this energy conversion may be a manifestation of mitochondrial damage, instead of a programmed defense mechanism^17^. In our current study, cisplatin impaired mitochondrial function and increased the production of pyruvate and lactate, indicating that cisplatin-induced a transition away from oxidative phosphorylation to aerobic glycolysis. ATP production was reduced during this process. Thus, this metabolic shift was a reduction in functional glycolysis related to increased expression of glycolytic enzymes. Moreover, the inhibitor of the rate-limiting enzyme for FAO exacerbated the disruption of OXPHOS in cisplatin-treated HK2 cells. This phenomenon was also observed in cisplatin treatment MPC2 knockdown cells which was verified with CPT repressor and siRNAs. Our results support the previous view that pharmacological activation of the FAO can be a promising target for AKI treatment through a tolerance mechanism^18^.

Glutamine, a nonessential amino acid, is a main respiratory fuel and an essential metabolic precursor for synthesizing key molecules^19^. Previous studies have shown that glutamine protects against IRI-induced AKI *in vivo*^20^. Also, glutamine improved cisplatin-induced cell apoptosis in renal tissues^21^. However, a large multicenter clinical trial showed an increased death rate in critically ill patients with renal dysfunction receiving parenteral glutamine supplementation^22^. Recently, it was reported that targeting T cell glutamine pathway can improve the outcomes of ischemic and nephrotoxic AKI^23^. Our present findings demonstrated that the selective glutaminase inhibitor did not promote the disruption of OXPHOS in cisplatin-treated HK2 cells. Thus, glutamine oxidation might be dispensable for the survival of HK2 cells under both normal and cisplatin conditions.

The MPC complex is responsible for transporting pyruvate into mitochondria and facilitating the transition from glycolysis to oxidative phosphorylation^24^. Pyruvate-driven ATP production by oxidative phosphorylation in mitochondria is a central feature of kidney metabolism. Our previous study demonstrated that MPC1 and MPC2 were decreased and related to renal function in diabetic nephropathy^25^. Our data showed that MPC2 was significantly reduced in renal tubules in ATN patients and the cisplatin treatment model both *in vivo* and *in vitro*. Additionally, MPC2 has a prosurvival effect on cisplatin-induced AKI. MPC1 and MPC2 are not the same although structurally and functionally similar. A recent study showed MPC1 disruption increased antioxidant defense systems, enhanced the metabolic shift to glycolysis, and prevented AKI induced by rhabdomyolysis^26^. The function of MPC1 in brain diseases is still controversial. It was reported that the inhibition of MPC1 was anti-inflammatory and neuroprotective in the animal model of Alzheimer's Disease^27^. However, MPC1 may exert neuroprotective effects in the cortex with ischemia by regulating calcium homeostasis, oxidative stress, mitochondrial function, and autophagy^12^.

Mitochondria are the primary site where cisplatin damages proximal tubular epithelial cells^28^. Abnormal MPC1 can induce mitochondrial dysfunction^29^. Consistently, our study found that MPC2 could ameliorate mitochondrial respiratory chain abnormalities and restore mitochondrial structure and ATP production. MPC-mediated pyruvate flux into mitochondria is critical for oxidative metabolism and drives ATP generation^30^. Interestingly, it was reported that pyruvate exerted anti-inflammatory and anti-oxidant activity. Pyruvate administration protected against AKI by inducing the anti-inflammatory HO-1 and IL-10 expression^31^. Both inflammation and oxidative stress have been implicated in cisplatin nephrotoxicity^32^. Therefore, the protective effect of MPC-mediated pyruvate metabolism on renal tubular cell damage might involve more complex mechanisms. Moreover, a previous study found that inhibiting MPC or supplementing exogenous lactate led to increased chromatin accessibility together with overall chromatin remodeling of prostate epithelial cells lineage-specific transcription^33^. Future studies will be essential to clarify the underlying mechanisms.

Artemether is extracted from artemisinin and is widely used in antimalarial treatment. Artemisinin-based drugs have diverse biological activities, such as antifungal, antibacterial, antiviral, and anticancer properties^34^. The effect of artemether varies in different cell types. Like other active components of Chinese traditional medicine, artemether is more toxic for cancer cells than normal cells^35^. A previous study showed that artemether ameliorated cardiac atrophy induced by doxorubicin via modulating mitochondrial function and autophagy^36^. Moreover, artemether ameliorated adriamycin-induced nephropathy by improving redox balance and mitochondrial function in mice^37^. Consistently, our current study showed that artemether protected against cisplatin-induced AKI by modulating mitochondrial function. This protective role of artemether might be related to targeting MPC2 as it was declared previously^14^. More research is needed to comprehensively reveal the clear mechanisms of mitochondrial regulation and renal protection of artemether in the future.

In conclusion, our data verified the protective role of MPC in AKI from its capacity to regulate metabolic changes. Moreover, artemether as an MPC2 potential activator could mitigate AKI via MPC2-mediated pyruvate pathway.

## Materials and Methods

### Study approval

All experimental procedures regarding human tissue received approval from the Ethics Committee of the First Affiliated Hospital of Nanjing Medical University (2021-SR-398) and were performed in accordance with the rules for the Declaration of Helsinki. Signed informed consent was obtained from patients involved in this study. All animal experiments received approval from the Animal Care and Use Committee of Nanjing Medical University (2205035).

### Reagents and antibodies

Cisplatin, UK5099, BPTES, Etomoxir, and Artemether were provided by Sigma-Aldrich (St. Louis, MO). anti-MPC2 and anti-cleaved caspase 3 were derived from CST (Danvers, MA, USA), anti-GAPDH provided by Wuhan Sanying Biotechnology. The secondary HRP-antibodies were obtained from Beijing Zhongshan Golden Bridge Biotechnology Co., Ltd.

### Patients

Kidney biopsies were obtained from 11 hospitalized participants with AKI who consented to research biopsies. The tissues in the control group were obtained from patients diagnosed with minimal change disease (MCD). The patient information was listed in Supplemental Table 1.

### Animals

Male C57BL/6J mice, 8-12 weeks old, were randomly assigned for the experiment. AKI models were established by intraperitoneal injection of cisplatin (30 mg/kg)^38^. For the therapeutic effect of artemether, mice were intraperitoneally injected with artemether (25 mg/kg) 1 h before modeling, followed by consecutive injection after modeling. Mice were sacrificed 3 days after the injection of cisplatin. The blood and kidney tissues were harvested.

### *In vivo* MPC2 knockin experiment

A mixture of 50 µL /DNase-free water, containing 50 μg per mouse MPC2 plasmid or the same amount of vector, and 50 μL of transfection reagent (Altogen Biosystems, Las Vegas, Nevada, USA) was administered by tail vein injection into the C57BL/6J mouse.

### Cell culture and treatment

Human proximal tubular epithelial cells (HK2 cells) were maintained in DMEM/F12 (Gibco, USA) plus 10% FBS, 1% penicillin/streptomycin at 37℃ with 5% CO_2_^39^. After pretreatment with 60 μM artemether, HK2 cells were given cisplatin (40 μM) for indicated times. HK2 cells were transfected with MPC2-targeted siRNAs or plasmids (addgene, USA) using Lipofectamine 2000 Reagent (Invitrogen, USA). The MPC2 siRNA sequences were as follows: forward 5'-3': GAG UCU GUU UGC UGU UAA UTT, reverse 5'-3': AUU AAC AGC AAA CAG ACU CTT.

### Renal histology

The paraffin-embedded renal tissue blocks were sliced into 2 μm sections for staining with H&E (hematoxylin-eosin) and PAS (periodic acid-Schiff) in line with standard protocols. Tubular damage score was valued by the percentage of tubular injury (0, normal; 1, <10%; 2, 10%-25%; 3, 25%-50%; 4, 50%-75%; and 5, 75%-100%)^40^.

### Kidney function

The serum creatinine (SCr) and blood urea nitrogen (BUN) levels were assessed by commercial kits as before^41^, following the manufacturer's protocols.

### Western blots

Western blotting was performed as previously described^42^. In brief, fresh renal tissues and cultured cells were lysed with RIPA buffer involving protease inhibitor cocktail (Sigma-Aldrich). After blocking, the polyvinylidene fluoride (PVDF) membranes were incubated overnight with primary antibody dilutions (MPC2, 1:1000; cleaved caspase 3, 1:1000; GAPDH, 1:10000, respectively). The quantification of band intensities were calculated by Image Lab Software (Bio-Rad, USA).

### Apoptosis

*In situ* cell death detection kit (Roche Diagnostics, German) was used for the TUNEL staining following the standard protocols. I*n vitro*, HK2 cells apoptosis was performed using Annexin V-FITC Apoptosis Detection Kit (BD Biosciences, USA) following the manufacturer's instructions. Flow cytometry was used to detect the apoptosis levels of cells within 1 h.

### Cell viability

Cells were seeded into 96-well plates. After treatment, 10ul CCK-8 (Dojindo, Japan) solution was added into cells followed by incubated for 2 h at 37 ℃. Cell viability was measured by detecting absorbance at 450nm.

### Immunohistochemistry

Immunohistochemical evaluation of proteins was performed on paraffin-embedded tissue sections (3 μm) as previously described^43^. The sections exposed to primary antibodies for MPC2 (1:100) were maintained at 4 °C overnight. After washing with PBS for three times, the sections were incubated with secondary antibodies and visualized using a quick immunohistochemistry MaxVision DAB kit (MXB Biotechnologies, China).

### Immunofluorescence

The renal sections were embedded in OCT compound (Tissue-Tek, Sakura Finetek Europe BV) after being fixed in 4% paraformaldehyde for 15 minutes. Frozen slices were incubated by the indicated primary antibodies maintaining 4 °C overnight. Then, the stained sections were incubated with fluorescence-conjugated secondary antibodies for one hour at 37 ℃. The acquisition of pictures was performed utilizing a microscope (Leica, Germany).

### Pyruvate and lactate levels

According to a prior study, pyruvate concentrations in cell culture media, serum and kidney tissue were examined by a pyruvate assay kit (Beijing Solarbio Science & Technology Co., Ltd.) in line with the manufacturer's protocols. Lactate concentrations were detected by lactate assay kit (Nanjing Jiancheng Bioengineering Institute) according to the manufacturer's instructions. Cellular or tissue protein levels were used for normalization.

### Mitochondrial respiration analysis

OCR were evaluated by Seahorse XF96 Analyzer (Agilent, USA) following standard protocol^4^. In brief, HK2 cells were planted in XF96 Cell Culture Microplates (2×10^4^ cells/well) for 24 h before the experiments. After treatment, cell medium was changed into Seahorse XF Assay medium. Subsequently, OCR was assessed at baseline and after the injection of different inhibitors: oligomycin (1.25 μM), FCCP (0.75 mM), rotenone (1 μM) and antimycin A (1 μM) mixture.

### Mitochondrial morphology

Mitochondrial fission *in vitro* was stained with MitoTracker Red. Following experimental treatments, HK2 cells were co-incubated with MitoTracker Red (200 nM ) for 20 minutes followed by washing three times with basic culture medium. The mitochondria were observed by laser scanning confocal microscopy^44^.

### ATP generation

ATP production was tested by firefly luciferase-based ATP assay kit (Beyotime Institute Biotechnology, Nanjing, China) following the standard instruction. Briefly, cells were lysed with lysis buffer, superior solutions were obtained after centrifugation. Add 100ul ATP working solution into tubes and leave at room temperature for 5 minutes. Subsequently, the samples or standards were added into the tubes and mixed quickly. The value of ATP was detected by a luminometer. Finally, ATP concentration was normalized to protein content.

### Statistical analysis

All data were shown as means ± standard error of mean (SEM) and were performed with one way-ANOVA followed by Bonferroni tests by using SPSS version 22.0 for Windows. Correlation analyses were conducted with Pearson's correlation test. *P* values less than 0.05 indicated statistical significance.

## Supplementary Material

Supplementary methods, figures and table.

## Figures and Tables

**Figure 8:**
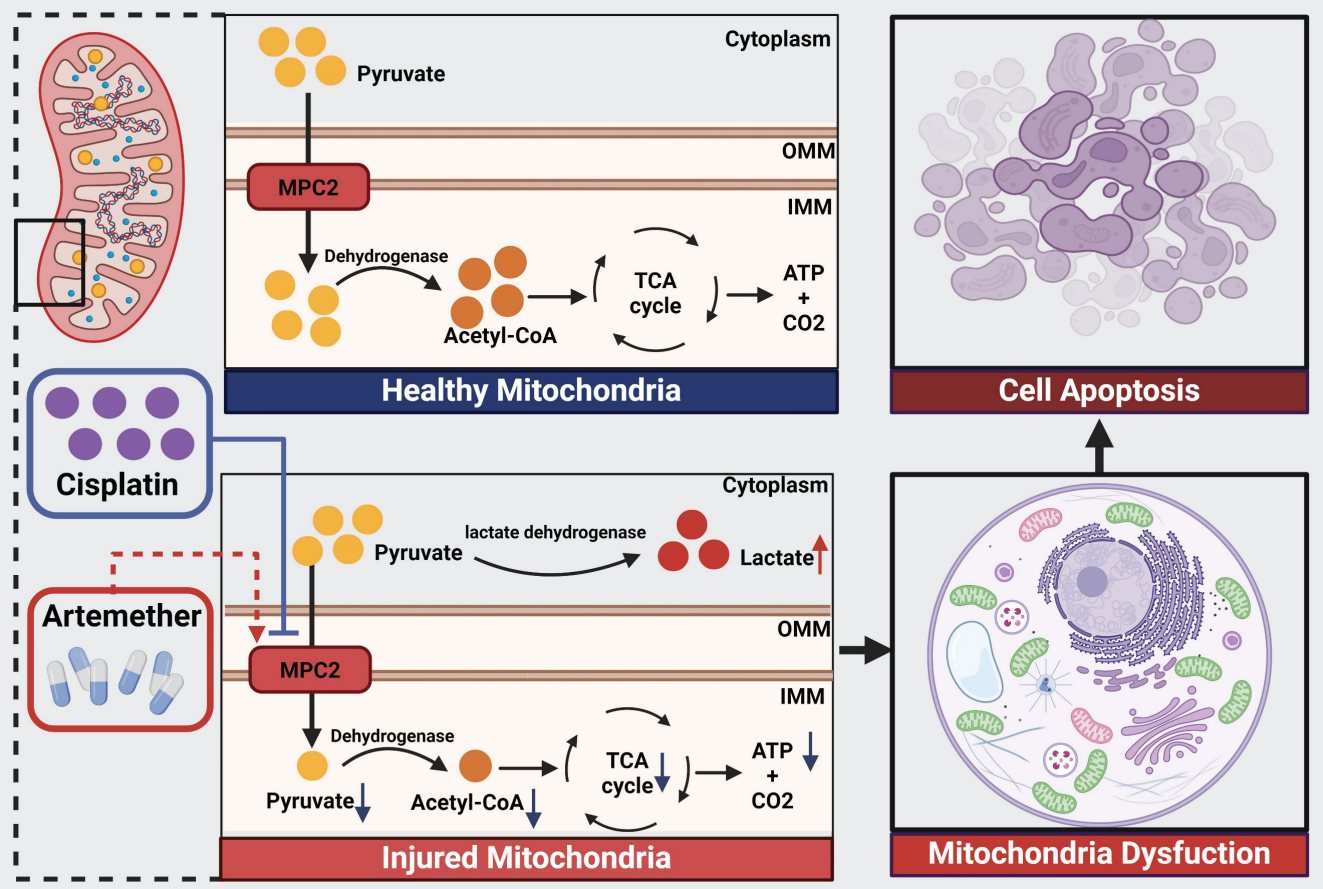
** Schematic illustration of the mechanism of MPC2 in AKI.** MPC2 was reduced in renal tubular cells during cisplatin treatment, where it disrupted the transport of the pyruvate from the cytosol to the mitochondria, leading to metabolic reprogramming way from fatty acid oxidation-driven oxidative phosphorylation to glycolysis with ensuing tubular cell death. Artemether, an MPC2 potential activator, could prevent AKI via regulating MPC2-mediated pyruvate metabolism.
