# Supplementary material for: Mitochondrial pyruvate carrier 2 mitigates acute kidney injury via sustaining mitochondrial metabolism
Source: Int J Biol Sci. 2024 Aug 19;20(11):4551–65. doi: 10.7150/ijbs.98627 (PMC11380453; doi:10.7150/ijbs.98627)
Supplement: Supplementary file 1 — Supplementary methods, figures and table. [file ijbsv20p4551s1.pdf]

Supplementary methods

Gene expression datasets

Microarray and normalized high throughput sequencing data were downloaded from Gene Expression Omnibus (GEO) database (http://www.ncbi.nlm.nih.gov/geo). The GSE52004 dataset included data on 6 samples from mice with 3 sham surgery and 3 bilateral ischemic reperfusion injury (24h) on the GPL6246 platform ([MoGene-1_0-st] Affymetrix Mouse Gene 1.0 ST Array [transcript (gene) version]). The GSE106993 dataset contained data on kidney tissues from mice with 4 intraperitoneal cisplatin (72h) and 4 vehicle (sodium chloride 0.9%) injection on the GPL21103 platform (Illumina HiSeq 4000 (Mus musculus)). For GSE52004 dataset, the "Affy" package in R (https://www.r-project.org/) software was used to perform background correction and normalization. The level of gene expression changes in GSE52004 and GSE106993 datasets were identified by the "limma" package in R.

Single Cell RNA Sequencing Data Acquisition and Processing

Acute kidney injury models scRNA-seq data were downloaded from the Gene Expression Omnibus (GEO) database (http://www.ncbi.nlm.nih.gov/geo). The GSE139506 dataset included data on 2 samples from mice with 1 I/R 24h model and 1 sham operated control on the GPL17021 platform (Illumina HiSeq 2500 (Mus musculus)). Cisplatin induced AKI model GSE197266 scRNA-seq dataset included data on 4 samples from mice with 2 cisplatin treated and 2 control on the GPL24247 platform (Illumina NovaSeq 6000 (Mus musculus)). "DoubletFinder" was used to remove the potential doublets. "Seurat v5" was used to standardize the expression of

filtered samples. Cells were filtered out with the threshold of the ratio of mitochondrial genes ≤50%. Genes expressed in >5 cells and cells with at least 200 genes at most 5000 were retained. “CCA” data integration method was used to integrate different seurat objects.

Dimensionality Reduction and Cell Annotation

The integrated data proceeded with principal component analysis (PCA), and the top 30 PCs were reduced by the UMAP algorithm to obtain principal clusters. Then the proximal tubule cells were labeled and clustered.

Differentially Expressed Genes Identification

The “FindMarkers” function in Seurat was used to compare the gene expression between cisplatin or I/R induced AKI model and healthy control groups in different celltypes. The differently expressed genes were screened with the restriction of adjust P value < 0.05 and $\log2 |\text{fold change}| > 0.5$.

Supplemental Table

Table S1. The basic information and diagnosis of renal biopsy specimens.

	Gender	Age, years	Pathologic diagnosis	Serum creatinine, umol/L	BUN, mmol/L
1	Female	37	MCD	59.1	3.90
2	Female	37	MCD	57.3	5.00
3	Male	29	MCD	80.6	4.80
4	Male	29	MCD	83.0	5.25
5	Male	32	MCD	74.3	4.25
6	Male	24	MCD	65.2	4.56
7	Female	27	MCD	82.8	4.30
8	Male	29	MCD	57.3	4.72
9	Male	27	MCD	68.0	4.72
10	Female	57	MCD	49.2	6.22
11	Female	28	MCD	47.9	7.39
12	Male	77	ATN	747.8	33.93
13	Male	62	ATN	488.6	14.34
14	Female	65	ATN	201.9	20.65
15	Male	20	ATN	211.6	9.00
16	Female	49	ATN	469.7	13.19
17	Male	72	ATN	228.8	13.90
18	Female	44	ATN	264.5	25.50
19	Male	49	ATN	705.5	59.40
20	Female	54	ATN	386.5	27.39
21	Female	55	ATN	235.9	9.90
22	Female	39	ATN	477.6	18.67

Supplementary Figure Legends

Supplementary Figure S1. (A) Schematic representation of the metabolic pathways. (B) Experimental scheme of mitochondrial stress test. (C-E) Change of OCR after the addition of inhibitors (5 μM UK-5099, 8 μM etomoxir, or 6 μM BPTES) was calculated with or without cisplatin treatment. (F) Quantification of basal mitochondrial respiration, (G) maximal mitochondrial respiration, and (H) ATP production. Data are expressed as the means ± SEM ($n \geq 5$). ∗$P < 0.05$ versus control. #$P < 0.01$ versus cisplatin treatment group.

Supplementary Figure S2. (A) Heatmap of the key components in the fatty acid oxidation pathway and pyruvate metabolism in control mice and mice after ischemia reperfusion injury (IR). Expression of MPC1 (B), MPC2 (C), CPT1a (D), CPT1b (E), CPT1c (F) and CPT2 (G) genes in normal and IR kidney in violin plots. (H) Heatmap of the key components in the fatty acid oxidation pathway and pyruvate metabolism in control mice and mice after cisplatin treatment. Expression of MPC1 (I), MPC2 (J), CPT1a (K), CPT1b (L), CPT1c (M) and CPT2 (N) genes in normal and cisplatin treatment kidney in violin plots. ∗$P < 0.05$ versus control.

Supplementary Figure S3. ScRNA-seq profile of AKI models and the expression of MPC2 in different cells and groups. (A-B). UMAP plots of GSE197266 cisplatin kidney datasets and GSE139506 IRI kidney datasets. LOH, loop of Henle; DCT, distal convoluted tubule; CD-PC, principle cells of collecting duct; CD-IC, intercalated cells of collecting duct; PT, proximal tubule; Endo, endothelial cells; Podo, podocytes; Myofibro, myofibroblasts; Mac, macrophages; B_cell, B cells;

T_cell, T cells; Stromal, stromal cells; Mixed_identity, cells expressing markers of different renal cell types; Cell cycle prox, cells upregulating cell cycle markers, such as Mki67. (C). The expression MPC2 between different cells in cisplatin and IRI AKI models. (D). UMAP displaying the clustering of proximal tubular cells in 2 datasets. (E). The expression of MPC2 between different group of cisplatin and IRI AKI models in PT cells. (F). The expression of MPC2 between different type of PT cells in cisplatin and IRI AKI models.

Supplementary Figure S4. (A-C) Change of OCR after the addition of inhibitors (5 μM UK-5099, 8 μM etomoxir, or 6 μM BPTES) in MPC2 knockdown HK2 cell with cisplatin treatment. (D) Quantification of basal mitochondrial respiration, (E) maximal mitochondrial respiration, and (F) ATP production. (G) OCR were measured in HK2 cells transfected with scramble, CPT1, or CPT2 siRNA and followed by cisplatin treatment. (H) Quantification of mitochondrial stress test. (I) Western blot detection of CPT1a in HK2 cells with cisplatin treatment at different points in time. (J) Densitometric analysis of (I). (K) Western blot detection of CPT1a in HK2 cells with cisplatin treatment at different doses. (L) Densitometric analysis of (K). Data are expressed as the means ± SEM ($n \geq 5$). *$P < 0.05$ versus control.

Figure S1

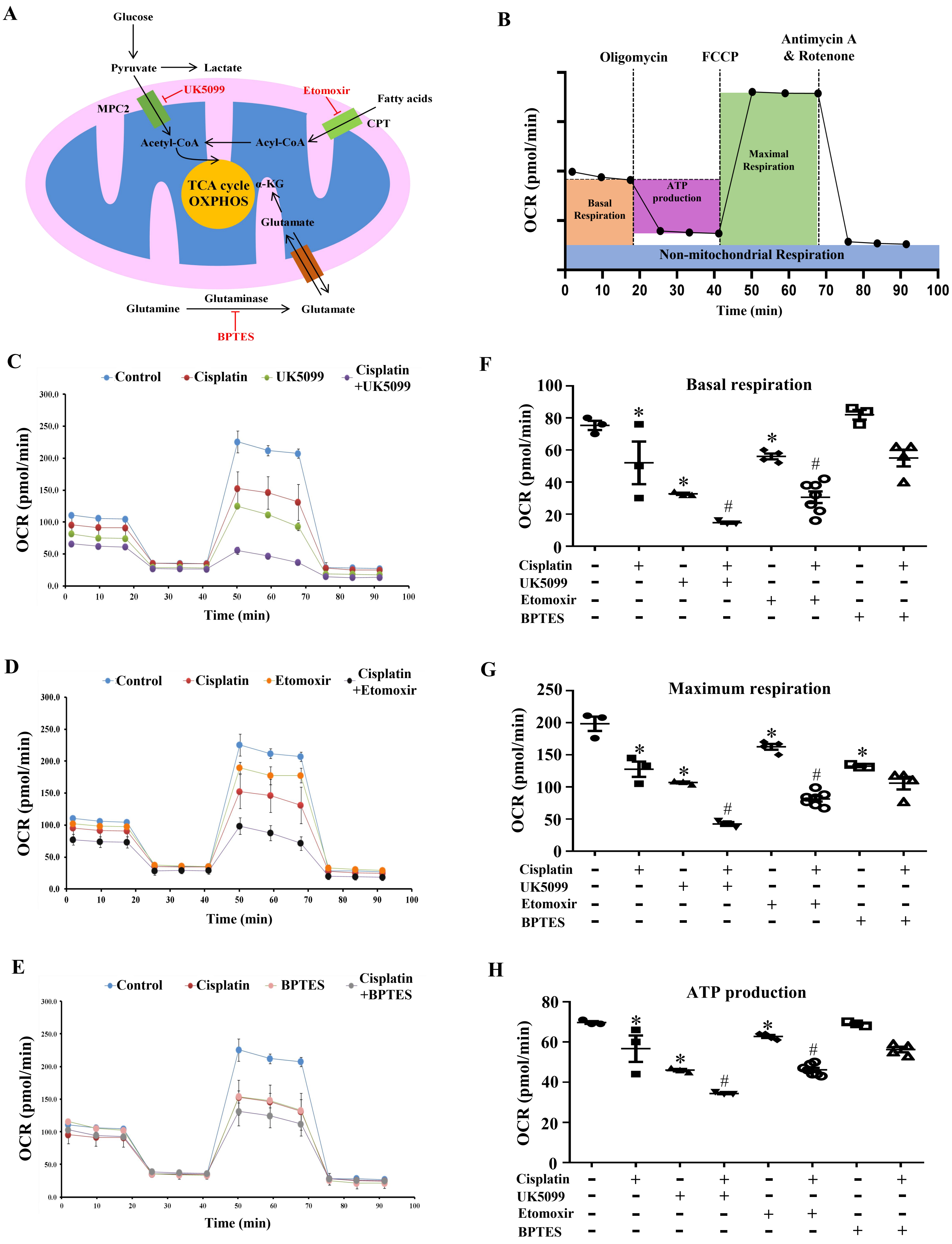
A
Glucose
Pyruvate
Lactate
UK5099
Etomoxir
Fatty acids
MPC2
CPT
Acetyl-CoA
Acyl-CoA
TCA cycle
OXPHOS
α-KG
Glutamate
Glutamine
Glutaminase
Glutamate
BPTES
B
Oligomycin
FCCP
Antimycin A
& Rotenone
OCR (pmol/min)
Basal
Respiration
ATP
production
Maximal
Respiration
Non-mitochondrial Respiration
Time (min)
C
Control
Cisplatin
UK5099
Cisplatin
+UK5099
OCR (pmol/min)
Time (min)
D
Control
Cisplatin
Etomoxir
Cisplatin
+Etomoxir
OCR (pmol/min)
Time (min)
E
Control
Cisplatin
BPTES
Cisplatin
+BPTES
OCR (pmol/min)
Time (min)
F
Basal respiration
OCR (pmol/min)
Cisplatin
UK5099
Etomoxir
BPTES
G
Maximum respiration
OCR (pmol/min)
Cisplatin
UK5099
Etomoxir
BPTES
H
ATP production
OCR (pmol/min)
Cisplatin
UK5099
Etomoxir
BPTES

Figure S2

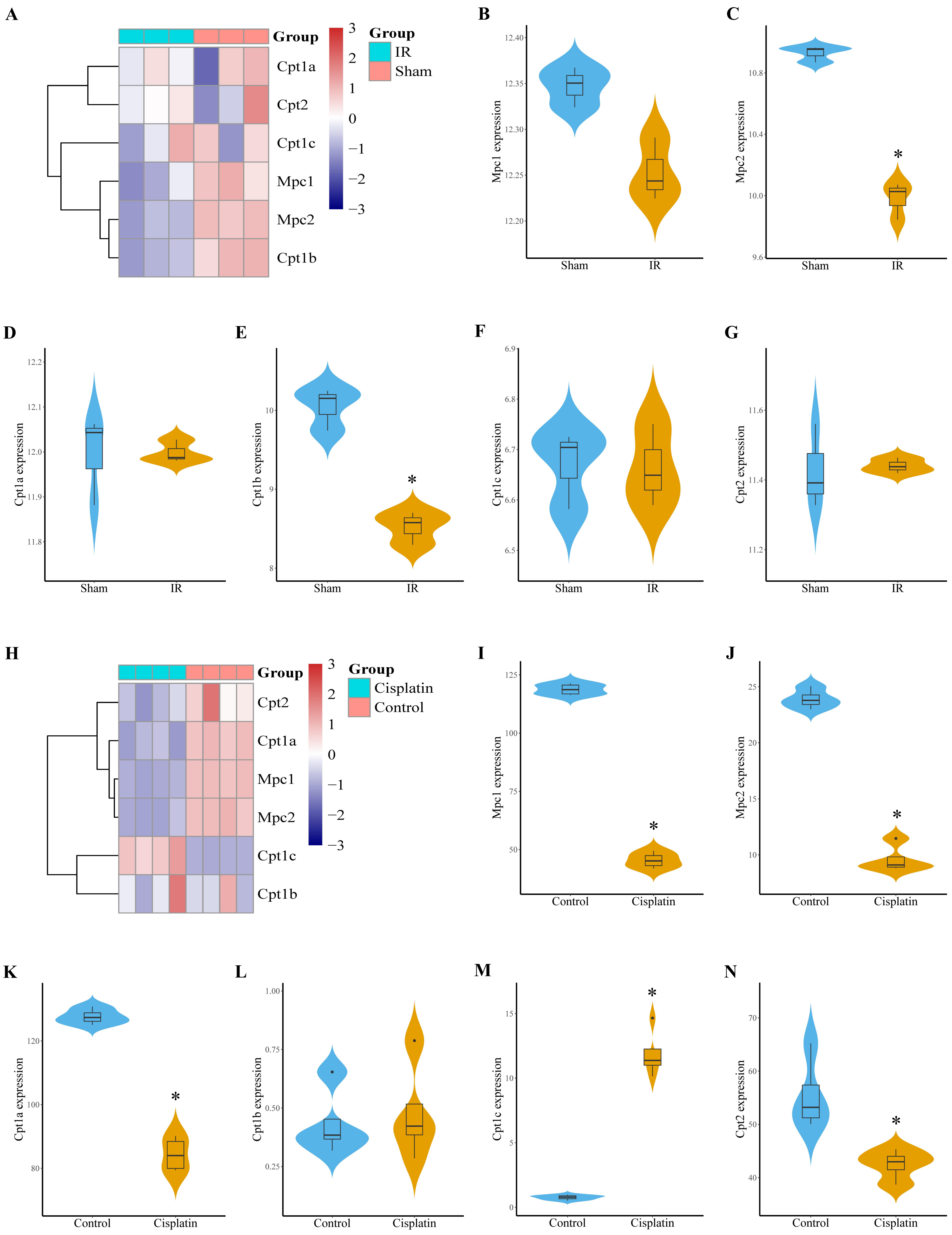
A
Group
Cpt1a
Cpt2
Cpt1c
Mpc1
Mpc2
Cpt1b
3
2
1
0
−1
−2
−3
Group
IR
Sham
B
Mpc1 expression
12.40
12.35
12.30
12.25
12.20
Sham
IR
C
Mpc2 expression
10.8
10.4
10.0
9.6
*
Sham
IR
D
Cpt1a expression
12.2
12.1
12.0
11.9
11.8
Sham
IR
E
Cpt1b expression
10
9
8
*
Sham
IR
F
Cpt1c expression
6.9
6.8
6.7
6.6
6.5
Sham
IR
G
Cpt2 expression
11.6
11.4
11.2
Sham
IR
H
Group
Cpt2
Cpt1a
Mpc1
Mpc2
Cpt1c
Cpt1b
3
2
1
0
−1
−2
−3
Group
Cisplatin
Control
I
Mpc1 expression
125
100
75
50
*
Control
Cisplatin
J
Mpc2 expression
25
20
15
10
*
Control
Cisplatin
K
Cpt1a expression
120
100
80
*
Control
Cisplatin
L
Cpt1b expression
1.00
0.75
0.50
0.25
Control
Cisplatin
M
Cpt1c expression
15
10
5
0
*
Control
Cisplatin
N
Cpt2 expression
70
60
50
40
*
Control
Cisplatin

Figure S3

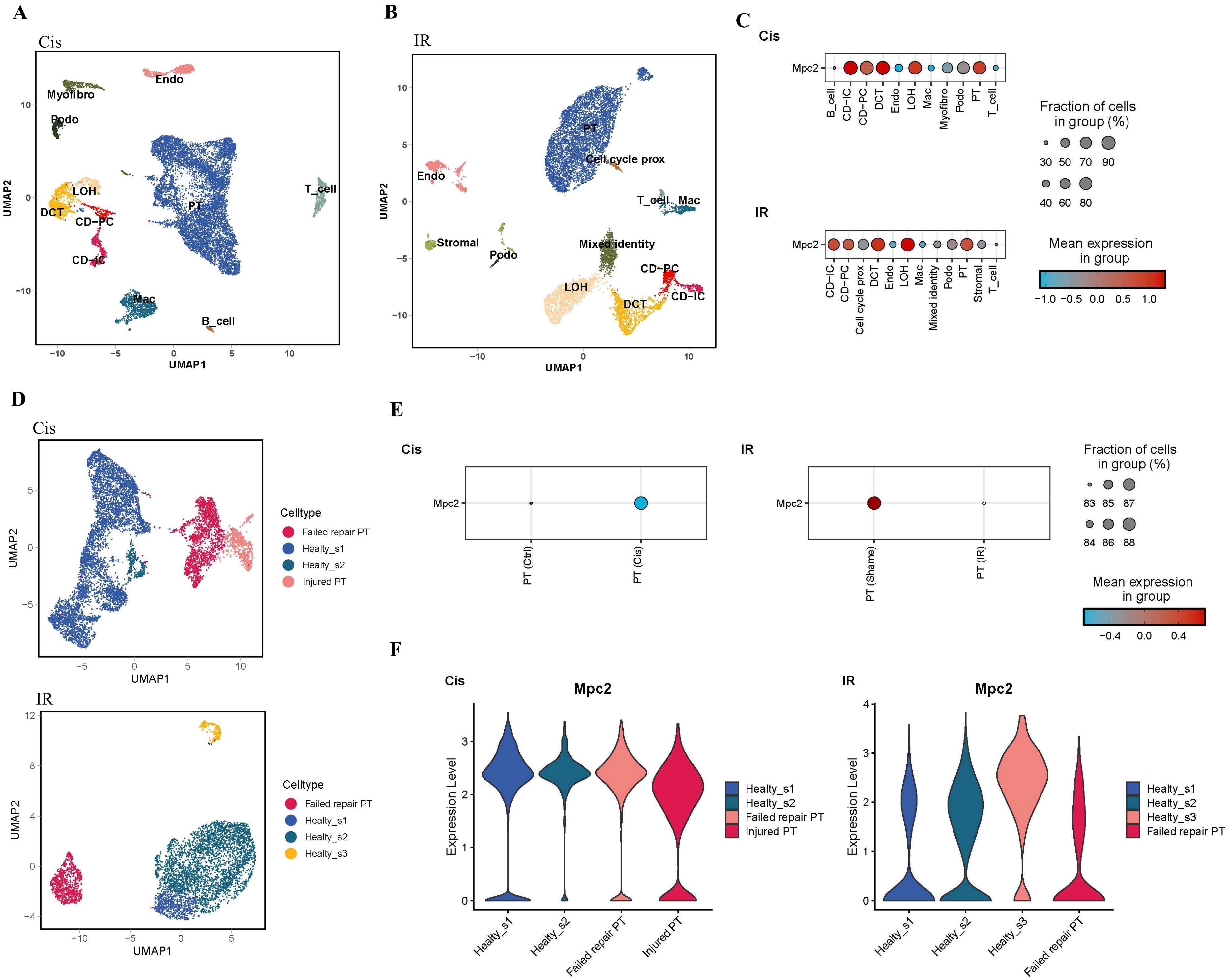
A
Cis
Myofibro
Endo
Podo
LOH
DCT
CD−PC
CD−IC
PT
T_cell
Mac
B_cell
UMAP1
UMAP2
B
IR
PT
Cell cycle prox
Endo
T_cell
Mac
Stromal
Podo
Mixed identity
CD−PC
CD−IC
LOH
DCT
UMAP1
UMAP2
C
Cis
Mpc2
B_cell
CD−IC
CD−PC
DCT
Endo
LOH
Mac
Myofibro
Podo
PT
T_cell
IR
Mpc2
CD−IC
CD−PC
Cell cycle prox
DCT
Endo
LOH
Mac
Mixed identity
Podo
PT
Stromal
T_cell
Fraction of cells in group (%)
30 50 70 90
40 60 80
Mean expression in group
−1.0 −0.5 0.0 0.5 1.0
D
Cis
Celltype
Failed repair PT
Healty_s1
Healty_s2
Injured PT
UMAP1
UMAP2
IR
Celltype
Failed repair PT
Healty_s1
Healty_s2
Healty_s3
UMAP1
UMAP2
E
Cis
Mpc2
PT (Ctrl)
PT (Cis)
IR
Mpc2
PT (Shame)
PT (IR)
Fraction of cells in group (%)
83 85 87
84 86 88
Mean expression in group
−0.4 0.0 0.4
F
Cis
Mpc2
Expression Level
Healty_s1
Healty_s2
Failed repair PT
Injured PT
IR
Mpc2
Expression Level
Healty_s1
Healty_s2
Healty_s3
Failed repair PT

Figure S4

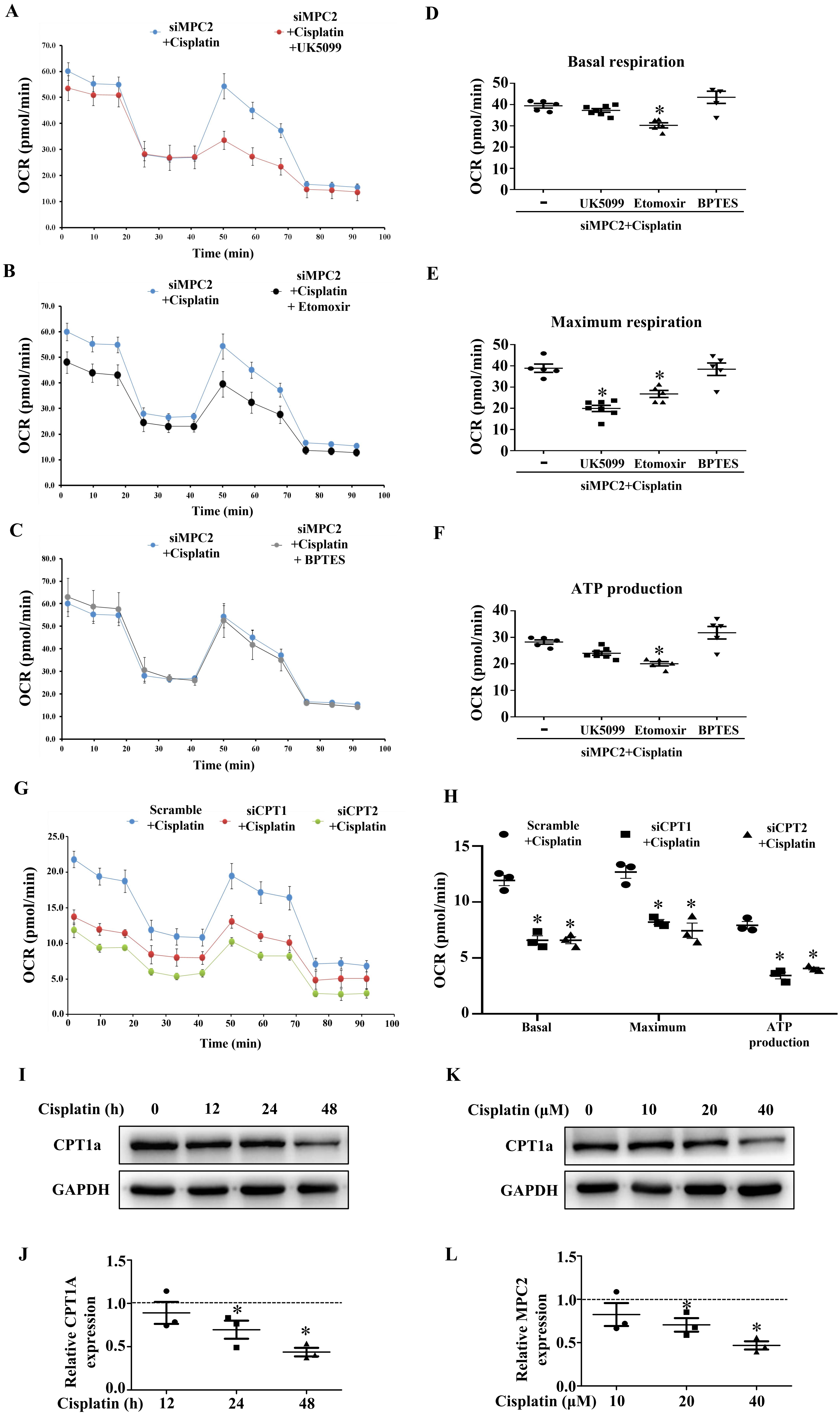

A
siMPC2 +Cisplatin
siMPC2 +Cisplatin +UK5099
OCR (pmol/min)
Time (min)
B
siMPC2 +Cisplatin
siMPC2 +Cisplatin + Etomoxir
OCR (pmol/min)
Time (min)
C
siMPC2 +Cisplatin
siMPC2 +Cisplatin + BPTES
OCR (pmol/min)
Time (min)
D
Basal respiration
OCR (pmol/min)
- UK5099 Etomoxir BPTES
siMPC2+Cisplatin
E
Maximum respiration
OCR (pmol/min)
- UK5099 Etomoxir BPTES
siMPC2+Cisplatin
F
ATP production
OCR (pmol/min)
- UK5099 Etomoxir BPTES
siMPC2+Cisplatin
G
Scramble +Cisplatin
siCPT1 +Cisplatin
siCPT2 +Cisplatin
OCR (pmol/min)
Time (min)
H
Scramble +Cisplatin
siCPT1 +Cisplatin
siCPT2 +Cisplatin
OCR (pmol/min)
Basal
Maximum
ATP production
I
Cisplatin (h) 0 12 24 48
CPT1a
GAPDH
K
Cisplatin (μM) 0 10 20 40
CPT1a
GAPDH
J
Relative CPT1A expression
Cisplatin (h) 12 24 48
L
Relative MPC2 expression
Cisplatin (μM) 10 20 40
